# Non-enhancing Glioblastoma: A Case Report

**DOI:** 10.7759/cureus.41153

**Published:** 2023-06-29

**Authors:** Dahye Park, Ninh Doan

**Affiliations:** 1 Department of Medicine, University of Alabama at Birmingham School of Medicine, Birmingham, USA; 2 Department of Neurosurgery, Baptist Medical Center South, Montgomery, USA

**Keywords:** neurology case report, malignant brain tumor, non-enhancement, brain mri, glioblastoma multiforme

## Abstract

Glioblastoma multiforme (GBM) is a malignant adult brain tumor that is visualized as an enhancing lesion on post-contrast magnetic resonance imaging (MRI). In this study, we present a rare case of non-enhancing GBM that required histopathological examination for a definitive diagnosis. Our findings emphasize the critical role of biopsy in the diagnosis and treatment of GBMs, particularly in cases of non-enhancing lesions on MRI.

## Introduction

Glioblastoma multiforme (GBM) is an aggressive adult brain tumor. With a median survival of 15 months, a timely and accurate diagnosis of GBM is crucial for effective treatment and patient outcomes [1]. The current standard diagnosis for GBM is computed tomography (CT) or magnetic resonance imaging (MRI), with characteristic MRI findings including hypointensity on T1-weighted imaging and hyperintensity on T2-weighted imaging. Moreover, malignant gliomas such as GBMs are visualized as enhancing lesions on post-contrast images [2]. However, pathologically confirmed GBMs may show atypical MRI findings, making an accurate diagnosis of GBM more challenging. In this case report, we present a rare case of non-enhancing GBM, highlighting the importance of a comprehensive diagnostic approach to the management of GBM.

## Case presentation

**Figure 1:**
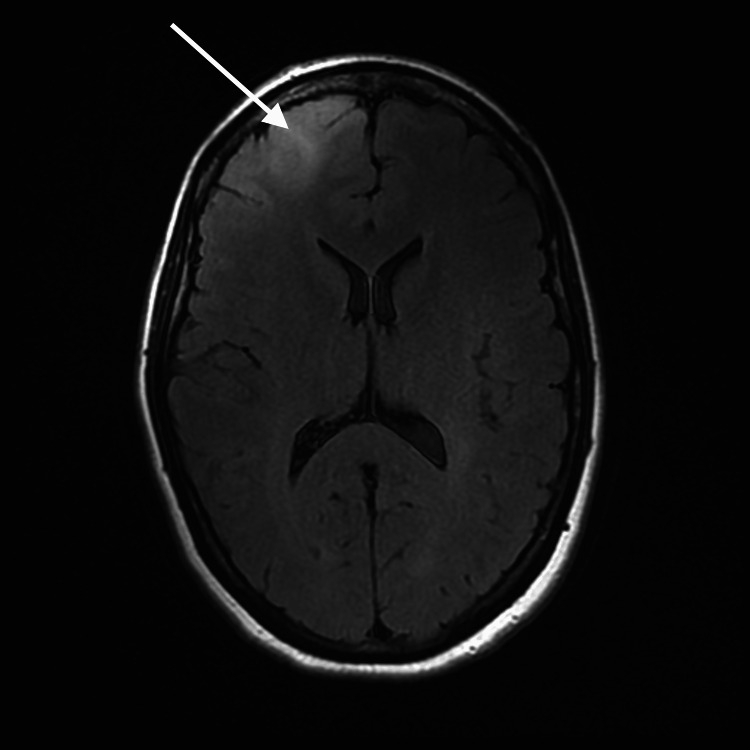
Axial T1 FLAIR demonstrating signal abnormality in the right frontal lobe (arrow) FLAIR: fluid-attenuated inversion recovery

**Figure 2:**
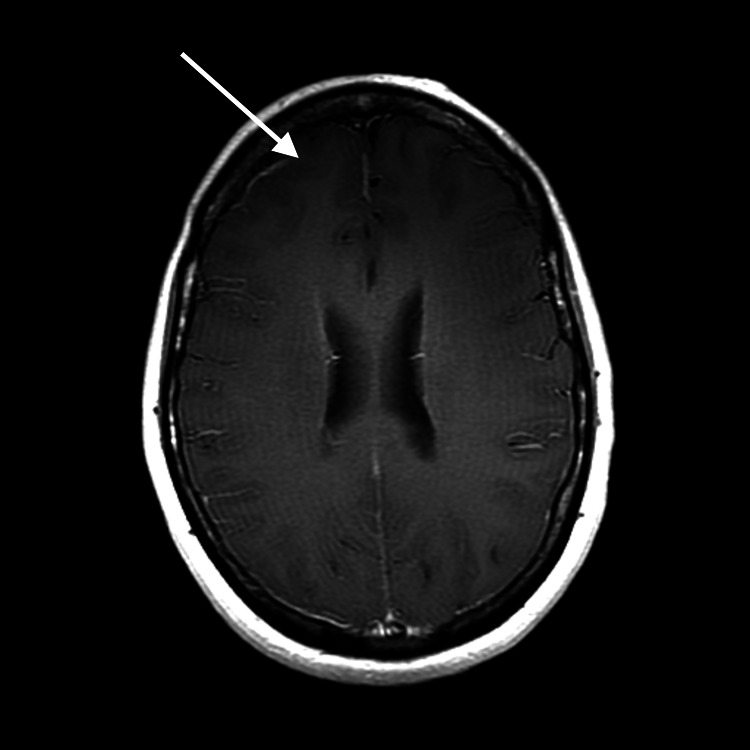
Lack of tumor enhancement (arrow) on post-contrast axial T1 MRI MRI: magnetic resonance imaging

A 48-year-old female presented to the emergency department (ED) with a one-month history of worsening confusion and headache. In the ED, the patient experienced a generalized tonic-clonic seizure and was promptly treated with lorazepam and levetiracetam. A cranial computed tomography (CT) showed no acute findings, but further examination with brain magnetic resonance imaging (MRI) revealed a T1 fluid-attenuated inversion recovery (FLAIR) signal abnormality in the right frontal lobe, measuring 3.4 × 3 cm, without enhancement or restricted diffusion on post-contrast images as shown in Figure 1 and Figure 2. Since the MRI findings showed a lack of enhancement, the frontal lobe abnormality was suspicious for a low-grade glioma, multiple sclerosis, or inflammatory disease. Furthermore, the electroencephalogram showed an abnormality characterized by mild cerebral dysfunction in the right frontal region. Results of a fluoroscopy-guided lumbar puncture were unlikely to indicate multiple sclerosis or demyelinating disease, leading to the recommendation of a right craniotomy for tumor mass resection and biopsy.

**Figure 3:**
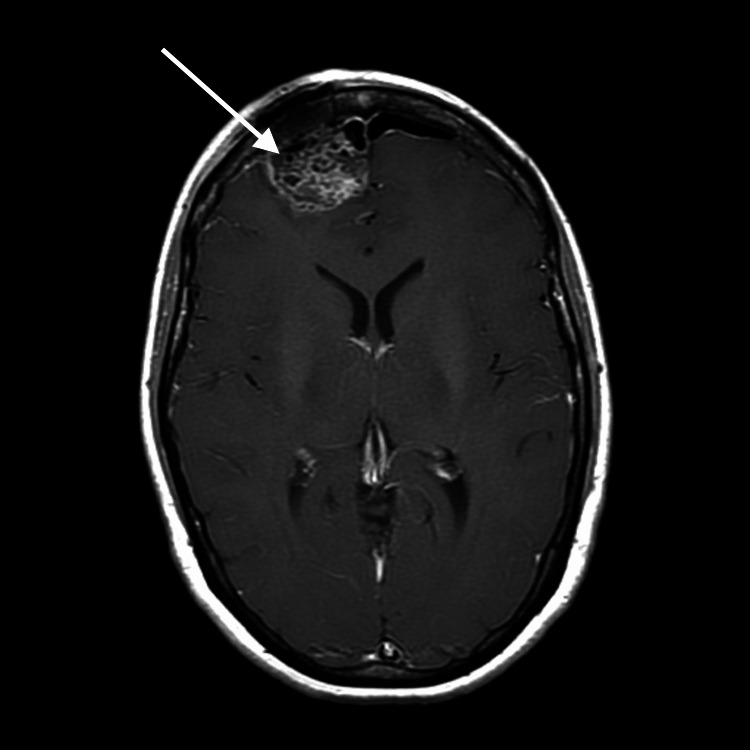
Post-contrast axial T1 MRI after craniotomy and resection of tumor (arrow) MRI: magnetic resonance imaging

Prior to surgery, a repeat brain MRI showed no changes from prior scans with the impression of a right frontal lobe lesion suspicious for low-grade glioma. Following surgery, histological examination confirmed grade IV methylated O6-methylguanine-DNA methyltransferase (MGMT), immunopositive for epidermal growth factor receptor (EGFR) and telomerase reverse transcriptase (TERT) promoter, and immunonegative for isocitrate dehydrogenase 1/2 (IDH1/2) with no definitive microvascular proliferation. Postoperative craniotomy MRI revealed blood products in the operative cavity and some subtle enhancement at the resection site as shown in Figure 3. The patient underwent chemotherapy, and the last follow-up brain MRI revealed no new disease progression.

## Discussion

Glioblastoma multiforme (GBM) is a highly malignant brain tumor and the most common form of adult brain cancer. Accurate diagnosis and characterization of GBMs are crucial for effective treatment and patient outcomes. Computed tomography (CT) and magnetic resonance imaging (MRI) are the standard diagnostic tools used for GBM diagnosis and monitoring of therapeutic response.

GBM is typically identified as an iso- to hypointense lesion on T1 and a hyperintense lesion on T2-weighted MRI scans. Moreover, high-grade gliomas such as GBM are often visualized as an enhancing lesion with central necrosis, while low-grade gliomas tend to show minimal contrast enhancement [2]. However, some cases reported visualization of GBM as a non-enhancing lesion on MRI with contrast images. Such reports highlighted the possibility of low-grade enhancement in GBM, and thus, the absence of enhancement in these tumors cannot always indicate low-grade malignancy [3-5]. Therefore, further evaluation and diagnostic approach is required to make a definitive diagnosis.

The presented case highlights the importance of histopathological examination in diagnosing GBM. Although the patient’s MRI initially suggested a non-enhancing lesion, raising the possibility of a low-grade glioma, the biopsy was necessary to confirm the diagnosis. Failure to obtain a biopsy may have resulted in misdiagnosis and ineffective treatment for the patient. Moreover, a study has reported that non-enhancing GBMs may share similar pathological features such as positive p53 and negative EGFR and IDH1 markers with nuclear pleomorphism and pseudopalisading necrosis [3]. This study suggests that certain pathological markers may play a role in the contrast enhancement of GBMs on MRI.

In summary, these findings emphasize the critical role of histopathological examination in the diagnosis and treatment of GBMs. Healthcare providers must be aware of the possibility of GBM, even in cases of non-enhancing lesions on MRI, and should recommend further evaluation with a biopsy for definitive diagnosis and proper treatment.

## Conclusions

MRI is an indispensable tool for the detection and evaluation of GBM. However, GBM may present as a non-enhancing lesion, which can be challenging in determining the grade and management of the tumor. Therefore, a comprehensive diagnostic workup, including histopathological examination, should be conducted to ensure an accurate diagnosis and effective treatment of GBMs.

## References

[1] Thakkar JP, Dolecek TA, Horbinski C, Ostrom QT, Lightner DD, Barnholtz-Sloan JS, Villano JL (2014). Epidemiologic and molecular prognostic review of glioblastoma. Cancer Epidemiol Biomarkers Prev.

[2] Abd-Elghany AA, Naji AA, Alonazi B (2019). Radiological characteristics of glioblastoma multiforme using CT and MRI examination. J Radiat Res Appl Sci.

[3] Utsuki S (2012). Glioblastoma without remarkable contrast enhancement on magnetic resonance imaging. Int J Clin Med.

[4] Moore-Stovall J, Venkatesh R (1993). Serial nonenhancing magnetic resonance imaging scans of high grade glioblastoma multiforme. J Natl Med Assoc.

[5] Cohen-Gadol AA, DiLuna ML, Bannykh SI, Piepmeier JM, Spencer DD (2004). Non-enhancing de novo glioblastoma: report of two cases. Neurosurg Rev.

